# ZHX2 deficiency enriches hybrid MET cells through regulating E-cadherin expression

**DOI:** 10.1038/s41419-023-05974-y

**Published:** 2023-07-17

**Authors:** Yan He, Qimin Zhang, Yuanhong Chen, Yingjian Wu, Yuan Quan, Weihua Chen, Jing Yao, Peijing Zhang

**Affiliations:** 1 National Engineering Research Center for Nanomedicine, Key Laboratory of Molecular Biophysics of Ministry of Education, College of Life Science and Technology, Department of Oncology, Tongji Hospital, Huazhong University of Science and Technology, Wuhan, China; 2 Stem Cell Laboratory, the Second Affiliated Hospital, Fujian Medical University, Quanzhou, China; 3 Cancer Center, Union Hospital, Tongji Medical College, Huazhong University of Science and Technology, Wuhan, China; 4 Institute of Radiation Oncology, Union Hospital, Tongji Medical College, Huazhong University of Science and Technology, Wuhan, China

**Keywords:** Metastasis, Breast cancer

## Abstract

Growing evidence indicates that the epithelial to mesenchymal (E/M) hybrid state plays a key role in tumorigenesis. Importantly, a hybrid mesenchymal to epithelial transition (MET) state in which individual cells express both epithelial and mesenchymal markers was recently identified in vivo, further strengthening the bonds between the hybrid EMT state and cancer progression. However, the role and the molecular mechanisms by which the hybrid MET state is maintained in triple-negative breast cancer cells (TNBC) remain elusive. Here, we find that loss of ZHX2 expression results in the hybrid MET phenotype in mesenchymal TNBC cells. Mechanistically, through directly binding to the CDH1 promoter, depletion of ZHX2 specifically reactivates expression of CDH1 encoding E-cadherin, an epithelial marker that is crucial for maintaining epithelial phenotype. Functionally, loss of ZHX2 expression enriches the hybrid MET cells and inhibits the migration and dissemination of TNBC cells or organoids, which could be reversed by restoration of E-cadherin. Moreover, depletion of ZHX2 suppresses lung metastasis in preclinical models of TNBC. In patients with TNBC, ZHX2 expression was amplified and negatively correlated with the expression of E-cadherin. These findings suggest that loss of ZHX2 promotes the hybrid MET state to impair TNBC progression.

## Background

Metastasis can be driven by the cellular plasticity—a bidirectional transition between epithelial and mesenchymal phenotypes—the EMT and MET [1]. Cells undergoing EMT lose cell adhesion, acquire migratory and invasive characteristics, and invade the basement membrane into blood vessels as circulating tumor cells (CTCs) [2]. These CTCs undergoing MET then relocalize in distant organs to form secondary tumors [1]. Elucidating the principles of this cellular plasticity holds promise for key clues to halting metastatic progression.

The EMT-mediated metastasis is mainly due to the loss of E-cadherin which is essential for maintaining adherent junctions between adjacent cells, thereby conferring physical integrity to epithelial cells [3, 4]. E-cadherin loss has been shown to enhance aggressiveness and dedifferentiation of many carcinomas and has been associated with increased metastasis in several epithelial tumor types [5]. Therefore, E-cadherin has been widely recognized as a suppressor of tumor metastasis. Some EMT-inducing transcription factors (EMT-TFs), such as ZEB1, ZEB2, Snail, Slug, and Twist1, repress the transcription of CDH1 encoding E-cadherin and play the key roles in metastasis [6]. Moreover, most of the EMT-TFs induce the expression of multiple genes associated with mesenchymal state and repress genes contributed to epithelial state [6]. It has been reported that the miR-34/Snail and the miR-200/ZEB exist a mutually inhibiting loop to control the balance between epithelial and mesenchymal states, in which intermediate levels of both miR-200 and ZEB can generate a hybrid epithelial/mesenchymal (hybrid E/M) phenotype [7, 8]. Notably, epithelial-mesenchymal plasticity is regulated by tissue-specific coupling of this loop with many other key players. With the identification of phenotypic stabilizing factors such as GRHL2 and OVOL2, EMT control has emerged with a degree of refinement that can interact with standard EMT control mechanisms to stabilize the hybrid state [9, 10]. Although it is well known about the molecular mechanisms that promote EMT in cancer cells, the molecular players that help cells maintain the hybrid MET phenotype by regulating the transcription of E-cadherin remain largely unknown.

Zinc fingers and homeoboxes 2 (ZHX2), a member of the ZHX protein family, is a nuclear-localized transcriptional repressor. ZHX2 is involved in a variety of pathological conditions, including cancer, atherosclerosis, and other metabolic-related diseases. The target genes negatively regulated by ZHX2 include alpha-fetoprotein (AFP), H19, and glypican-3 (GPC3) [11–13]. Studies have shown that ZHX2 plays a tumor suppressor role in hepatocellular carcinoma by transcriptional repressing the key cell cycle regulators, cyclin E and cyclin A [14]. In addition, ZHX2 expression is down-regulated in multiple myeloma, and is negatively correlated with tumor proliferation and invasion [15, 16]. ZHX2 is also a potential tumor suppressor gene for Hodgkin lymphoma [17]. Different from the above studies, the latest studies found that ZHX2 promotes tumor growth and migration of renal carcinoma [18] and activates HIF1α oncogenic signaling in triple-negative breast cancer [19]. Therefore, the abnormal expression of ZHX2 is associated with a variety of tumors and plays different roles in different tumors. Further studies on the role and mechanism of ZHX2 in different tumors will provide more potential therapeutic strategies for tumor treatment.

In the current study, we investigated the role of ZHX2 in determining the hybrid MET TNBC. In addition, we provided evidence for the binding motif of ZHX2 in the CDH1 promoter. ZHX2 deficiency promoted hybrid MET status, which could be reversed by ectopic expression of ZHX2. Furthermore, depletion of ZHX2 abolished TNBC metastasis in vivo. This study suggested that in triple-negative breast cancer, ZHX2 deletion promotes a hybrid MET state and attenuates metastasis by regulating E-cadherin transcription.

## Materials and methods

### Cell culture

SUM159 and HEK293T cells were cultured in Dulbecco’s modified Eagle’s medium (DMEM) supplemented with 10% fetal bovine serum (FBS). MDA-MB-231 was cultured in 10% FBS RPMI 1640. Normal breast MCF10A was cultured in DMEM/F12 containing 5% horse serum, 20 ng/ml EGF (Peprotech), 0.5 mg/ml hydrocortisone (Sigma #H-0888), 100 ng/ml cholera toxin (Sigma #C-8052), and 10 µg/ml insulin (Sigma #I-1882). The SUM159 cell line was from S. Ethier [20]. All other cell lines were obtained from ATCC. Cells were maintained at 37 °C in a 5% CO_2_ incubator.

### Plasmids, lentiviral shRNA and sgRNA vectors

pcDNA-3.1-HA-ZHX2(WT), pcDNA-3.1-HA-ZHX2(ZHX2 shRNA/sg1-resistant), Control shRNA, ZHX2 shRNA, ZHX2 sgRNA(1), ZHX2 sgRNA(2) were provided by Dr. Qing Zhang at University of North Carolina School of Medicine[18]. ZEB1 shRNA was previously described. pLenti-V5-ZHX2 (ZHX2 shRNA/sg1-resistant), pLenti-SFB-ZHX2 (WT), pMH-MYC-ZHX2 (WT), E-cadherin shRNA were constructed using standard molecular biology techniques. All plasmids were sequenced to confirm validity. Target sequences were as follows:

Control shRNA: AACAGTCGCGTTTGCGACTGG, ZEB1 shRNA: AGATTTACTGTGCTGTCCT, E-cadherin shRNA: CCGAGAGAGTTACCCTACATA, ZHX2 shRNA: CCGTAGCAAGGAAAGCAACAA, ZHX2 sgRNA(1): CATGATACGTGCGACCGTGT, ZHX2 sgRNA(2): GATCACCCCCGAGAACCACG.

### Virus production and infection

The HEK293T packaging cell line was used for lentiviral amplification. Viruses were collected at 48 h and 72 h after transfection, respectively. The virus infected target cells with 8 μg/ml polybrene after passing through a 0.45-µm filter. Subsequently, target cell lines were screened under appropriate antibiotics.

### Western blot analysis and antibodies

Lysis buffer (50 mM Tris pH 6.8, 1% SDS, and 10% glycerol) supplemented with complete protease inhibitor was used to harvest whole cell lysates. Proteins were separated on 7.5-15% SDS-polyacrylamide gel and blotted onto a nitrocellulose membrane (Bio-Rad). Membranes were probed with the specific primary antibodies, followed by peroxidase-conjugated secondary antibodies. The following antibodies were used: antibodies to ZHX2 (1: 1000, GeneTex, GTX112232, clone C1C3), E-cadherin (1: 2000, BD Transduction Laboratories, 610181), Vimentin (1: 1000, Cell Signaling Technology, 5741, clone D21H3), MYC-tag (1: 3000, Proteintech, 16286-1-AP), HSP90 (1: 3000, BD Transduction Laboratories, 610419, clone 68) and GAPDH (1: 10,000, ABclonal, A19056).

### Quantitative real-time PCR (RT-qPCR)

Total RNA was isolated from cells using TRIzol (Gibco/Invitrogen) and cDNA synthesis was done using the PrimeScript RT Master Mix (Takara, RR036A). Quantitative real-time polymerase chain reaction (PCR) experiments were performed with QuantiNova SYBR Green PCR Kit (QIAGEN, 208052) in triplicates. The relative expression of messenger RNA (mRNA) was quantified using the 2^–ΔΔCt^ method. Primer sequences are listed in Supplementary Table S3.

### Immunofluorescence (IF) staining

Cells were cultured in chamber slides overnight. After washing with PBS, cells were fixed and permeabilized with 4% PFA in PBS containing 0.1% Triton X-100 for 20 min at room temperature. Cells were then blocked for non-specific binding with 1% BSA in PBS at room temperature for 1 h. After extensive washing in PBS, cells were incubated with the E-cadherin antibody (1: 200) and the Vimentin antibody (1: 300) for 3 h at room temperature, followed by incubation with Alexa Fluor Plus 555 goat anti-rabbit IgG (1: 1000, Invitrogen, A32732) and Alexa Fluor Plus 488 goat anti-mouse IgG (1: 1000, Invitrogen, A32723) for 45 min at room temperature. Cover slips were mounted on slides using anti-fade mounting medium with DAPI. Immunofluorescence images were acquired on an Olympus FV3000 fluorescence microscope.

### Flow cytometric analysis

Flow cytometry analysis of TNBC cells was performed using the Fixation/Permeablization Kit (BD Biosciences, 554714) according to the manufacturer’s instructions with some modifications. Briefly, one million cells were stained with 5 µl of Vimentin-FITC (BD Pharmigen, 562338) and 5 µl E-cadherin-PE (BD Pharmigen, 562870). Samples were run on the CytoFLEX S (Beckman Coulter), and data analyzed using CytExpert software.

### Chromatin immunoprecipitation (ChIP)

ChIP assays were performed using the ChIP Assay Kit (Beyotime, P2078) according to the manufacturer’s instructions with some modifications. Briefly, HEK293T cells transiently transfected with SFB-ZHX2 or SUM159 cells were crosslinked with 1% formaldehyde at 37 °C for 10 min before stop buffer was added to terminate the reaction. After cell lysis, cross-linked chromatin was sheared by sonication with 10 s on/10 s off for 6 cycles. Pre-cleared DNA was then used for immunoprecipitation with 5 µl of ZHX2 antibody or rabbit control IgG (Cell Signaling Technology, 2729 S) at 4 °C overnight. For exogenous ZHX2, S-protein agarose (Millipore, 69704) was used to incubate with cell lysates overnight at 4 °C. For the input control, 1% of the sonicated pre-cleared DNA was saved and purified at the same time with the precipitated immune complex. Quantitative RT-PCR (qPCR) analysis was then performed using primers listed in Table S4.

### DNA-mediated pull-down assay and HPLC-MS/MS analysis

The DNA-mediated pull-down assay was conducted as Akiko Hata described [21], with some modifications. Briefly, cells were sonicated in HKMG buffer (10 mM HEPES, pH 7.9, 100 mM KCl, 5 mM MgCl_2_, 10% glycerol, 1 mM DTT, and 0.5% of NP-40) containing protease and phosphatase inhibitors. Cell debris was removed by centrifugation. Cell extracts or ZHX2 protein obtained by eukaryotic in vitro translation kit (Promega, L1170) were pre-cleared with Streptavidin Sepharose High Performance (Cytiva, 17511301) for 1 hr, then incubated with 1 µg of biotinylated double-stranded oligonucleotides and 10 µg of poly[d(I-C)] (Roche, 10108812001) or 10 µg competitor (P0) overnight at 4 °C. DNA-binding proteins were collected with streptavidin-agarose beads for 3 h at 4 °C, and washed with HKMG buffer, separated on a SDS-PAGE gel, and identified by Western blotting.

Biotinylated double-stranded CDH1 promoter oligonucleotides were used to precipitate proteins from HEK293T nuclear extracts and sent the mixture to HPLC-MS/MS for protein expression profiling. Nuclear and Cytoplasmic Protein Extraction kits were from YEASEN (#20126ES50). This assay was performed with the support of QLBio Biotechnology Co., Ltd (Beijing, China). Transcription factor prediction was from http://bioinfo.life.hust.edu.cn/AnimalTFDB/ [22]. See details in the supplemental information.

### Luciferase reporter assay

In order to test the relation between ZHX2 concentration and E-cadherin reporter activity, ZHX2 deletion SUM159 cells (1 × 10^6^ cells/48-well plate) were transiently transfected with different amount of HA-ZHX2 (0, 100, and 200 ng), the human CDH1 promoter constructs in pGL4basic (P0, P1, P2, and P3) and reporter plasmid pCMV-Renilla. Alternatively, CDH1 promoter constructs and pCMV-Renilla were co-transfected in MDA-MB-231 sublines using the same protocol. To determine the motif of ZHX2 binding to the CDH1 promoter, ZHX2 expression vector cotransfected in SUM159 cells together with wild type (P2) or mutant CDH1 promoter constructs. Forty-eight hours after transfection, luciferase assays were performed by Dual-Luciferase Reporter Assay System (Promega, E1960). The experiments were repeated in triplicate with similar results.

### Wound healing assay and Transwell assay

The wound healing assay was performed using the ibidi Culture-Insert 4 Well (ibidi, 80466) according to the manufacturer’s instructions. Cells (4 × 10^4^ SUM159 cells or 3 × 10^4^ for MDA-MB-231 cells) were seeded into each well and incubated for 24 h to achieve a confluent layer. After appropriate cell attachment, gently removed the Culture-Insert 4 Well by using sterile tweezers. Several regions were marked and photographed at 0 h, 9 h, 24 h, or 28 h after the scratches were made. Phase-contrast microscopy images were taken using a microscope, and data analyzed using ImageJ software.

Cell migration assay was performed in 24-well transwell plate with 8-mm polyethylene terephalate membrane filters (Corning, 3422) separating the lower and upper culture chambers. Briefly, SUM159 or MDA-MB-231 cells were plated in the upper chamber at 5 × 10^4^ cells per well in serum-free DMEM medium. The bottom chamber contained DMEM medium with 10% FBS. Cells were allowed to migrate for 24 h in a humidified chamber at 37 °C with 5% CO_2_. After the incubation period, removed cells that did not migrate through the pores by cotton swabs. Filters were fixed with methanol for 10 min and stained using 0.5% crystal violet for 20 min and photographed.

### Embedded TNBC cells 3D culture

MDA-MB-231 cells (1 × 10^4^ cells/96-well plate) were seeded into 100 µl of Collagen I (10 µg/ml, Solarbio, C8062), plated in 96-well Ultra-low adsorption culture plate and maintained 100 μl MDA-MB-231 complete media to each well. After seeding the cells, centrifuge the plate to allow the cells to accumulate at the bottom of the plate, thereby promoting uniform spheroid formation. Keep culture for 9 days and change medium every 3 days. Phase-contrast microscopy images were taken using a microscope, and data analyzed using ImageJ software.

### Mouse experiments

Female BALB/c nude mice (5-week old) were obtained from SHUBEILI Biotechnology Co., Ltd (Wuhan, China) and were randomly divided into indicated groups. The mice in the groups were intravenous inoculation of 3 × 10^6^ MDA-MB-231 cells or orthotopic injection of 1 × 10^7^ MDA-MB-231 cells. All mice were sacrificed 6 weeks after injection, and the lungs were removed, weighed, and fixed for further study. All animal studies were conducted according to the guidelines for the care and use of laboratory animals and were approved by the Institutional Animal Care and Use Committee, Huazhong University of Science and Technology (IACUC Number: 2923).

### Human samples and Immunohistochemistry (IHC)/Immunofluorescence (IF)

The TNBC tissue microarray (product number: ZL-Brc3N961) containing 48 pairs of TNBC specimens and the corresponding tumor-adjacent tissues used for IHC were obtained from Zhuolibiotech Co., Ltd (Shanghai, China). The paired metastatic tumors and primary tumors in human tissues used for IF were obtained from Union Hospital, Tongji Medical College. The current study was approved by the People’s Hospital of Tongxu County (Henan, China).The IHC/IF assay was performed and with the support of Servicebio Co., Ltd (Wuhan, China) or Biossci Co., Ltd (Wuhan, China) and analyzed by its Aipathwell software.

### Survival analysis

The patients’ survival rate was analyzed according to https://kmplot.com [23]. We chose TNBC patients as follows: Lymph node status: all, ER status: all, PGR status: all, HER2 status: all, KI67 status: all, Nottingham histologic grade: all, and PAM50 subtype: Basal-like. Finally, 309 TNBC patients were included in the overall survival analysis. ZHX2 expression was chosen as upper tertile expression.

### Statistical analysis

All statistical analysis was conducted using Prism 8.0 (GraphPad Software). All graphs depict mean ± standard deviation (SD) unless otherwise indicated. Statistical significances are denoted as ns (not significant; *p* > 0.05), *p < 0.05, **p < 0.01, ***p < 0.001, ****p < 0.0001. The numbers of experiments were noted in figure legends. To assess the statistical significance of a difference between two conditions, we used unpaired two-tail Student’s *t*-test. For experiments comparing more than two conditions, differences were tested by a one-way ANOVA followed by Dunnett’s or Tukey’s multiple comparison tests.

## Results

### ZHX2 deficiency enriches hybrid MET TNBC cells

**Fig. 1:**
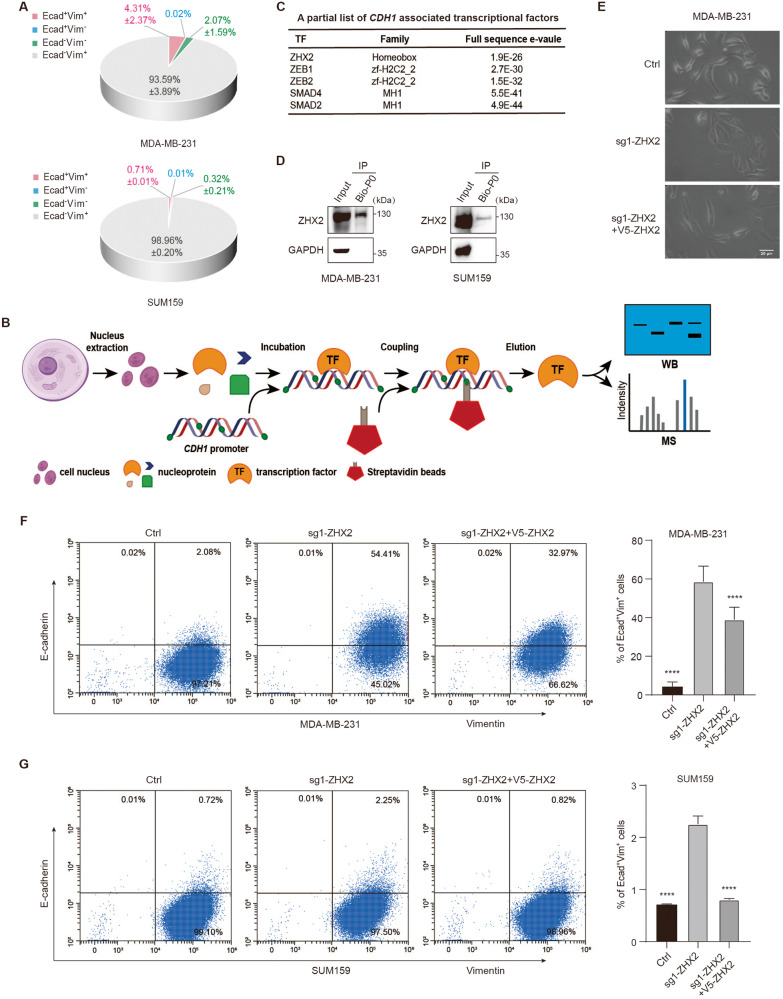
ZHX2 deficiency enriches hybrid MET TNBC cells. **A** Quantification of the proportion of epithelial marker E-cadherin (Ecad) and mesenchymal marker Vimentin (Vim) profiles by flow cytometry analysis in MDA-MB-231 (upper) and SUM159 (lower) cells. **B** Schema of DNA-mediated pull-down assay and HPLC-MS/MS analysis. Biotinylated double-stranded CDH1 promoter oligonucleotides were used to precipitate proteins from HEK293T nuclear extracts and sent the mixture to HPLC-MS/MS for protein expression profiling. **C** A partial list of transcription factors precipitated by the CDH1 promoter. **D** Cell lysates from MDA-MB-231 (left) or SUM159 (right) cells were precipitated with biotinylated full-length (P0) E-cadherin oligonucleotides followed by analysis of these complexes using anti-ZHX2 and anti-GAPDH antibodies. GAPDH is a negative control. **E** Morphological change of MDA-MB-231 cells infected with lentivirus encoding sg1-ZHX2 or control sgRNA (Ctrl), and rescued by ectopic expression of V5-ZHX2 in the knockout clones. Scale bar 20 μm. **F**, **G** Flow cytometry analysis of Vimentin (x-axis) against E-cadherin (y-axis) shows: **F** Ecad^+^/Vim^+^ population in MDA-MB-231 cells was increased from 2.08% in the control group to 54.41% in sg1-ZHX2 group while decreasing to 32.97% in sg1-ZHX2 + V5-ZHX2 group, **G** Ecad^+^/Vim^+^ population in SUM159 cells was increased from 0.72% in the control group to 2.25% in sg1-ZHX2 group while decreasing to 0.82% in sg1-ZHX2 + V5-ZHX2 cells. Quantitation is shown in the bar graphs. Error bars represent mean ± standard deviation (SD), one-way ANOVA. ****p < 0.0001.

Hundreds of EMT-related genes have been characterized and evaluated to quantify the EMT status of cells. Among all established markers, the Vimentin (Vim): E-cadherin (Ecad) expression ratio was shown to be the best approach for the assignment of various tumor cells into three phenotypes—epithelial, hybrid and mesenchymal [24]. Flow cytometry analysis of E-cadherin and Vimentin in mesenchymal-like TNBC cells clearly showed that there is 1–4.3% of hybrid MET cells in MDA-MB-231 and SUM159 cells (Fig. 1A and Supplementary Figure S1A).

To explore the molecular mechanism of maintaining the hybrid MET phenotype, we constructed biotinylated double-stranded CDH1 promoter oligonucleotides to precipitate its binding proteins from cellular nuclear extracts since Vimentin is stable in hybrid and mesenchymal cells (Fig. 1B). Protein expression profiling by HPLC-MS/MS identified 207 transcription factors, among them, ZEB1, ZEB2, SMAD2, and SMAD4 have been reported to be associated with EMT (Fig. 1C, Supplementary Table S1). SMADs promote transcriptional activation of EMT-TFs including ZEBs which could repress the transcriptional level of E-cadherin [6, 25]. Previous result showed that knockdown of ZEB1 or ZEB2 in genetically engineered mouse model organoids decreased distinct sets of mesenchymal genes without effect on epithelial gene expression such as E-cadherin [26]. We then examined E-cadherin expression in ZEB1 deficient mesenchymal TNBC cells. Consistently, ZEB1 was efficiently knocked down by ZEB1 shRNA [20], however, deficiency of ZEB1 could not reactivate E-cadherin expression to maintain the hybrid MET phenotype in TNBC cells (Supplementary Figure S1B, C). This result indicated that other transcription factors might involve in maintaining the hybrid MET phenotype by regulating E-cadherin expression. Among the identified transcriptional factors, ZHX2 stands out because it has been known that ZHX2 is a transcription factor associated with a variety of cancers [18]. Moreover, ChIPseq analysis indicated that ZHX2 has a binding peak in CDH1 promoter (Supplementary Figure S1D) [19]. In order to confirm the high-throughput results, we performed DNA-mediated pull-down assay, and immunoblotting results showed that the ZHX2 protein indeed bound to the CDH1 promoter in either MDA-MB-231 or SUM159 cells (Fig. 1D).

Next, we knocked out ZHX2 by infecting MDA-MB-231 cells with lentivirus encoding sgZHX2 or a control sgRNA (Ctrl), and reversed by ectopic expression of V5-ZHX2 in knockout clones (Supplementary Figure S1E). Interestingly, MDA-MB-231 knockout cells displayed a cobblestone-like appearance with tight cell-cell contact indicative of a MET phenomenon; whereas control cells or the ZHX2 expression restored cells (sg1-ZHX2 + V5-ZHX2) retained a spindle-like morphology with a scattered distribution (Fig. 1E). This morphological change induced by ZHX2 depletion indicated that ZHX2 played a major role in the regulation of the MET-like process. Moreover, flow cytometry analysis showed that the Ecad^+^/Vim^+^ population was remarkably enriched in sgZHX2 cells compared to control cells, which could be partially rescued after restoration of ZHX2 in knockout cells (Fig. 1F). Similar results were obtained in the SUM159 cells (Fig. 1G). These findings suggested that loss of ZHX2 skewed the TNBC cell population to a hybrid MET state.

### ZHX2 depletion upregulates the E-cadherin expression in TNBC

**Fig. 2:**
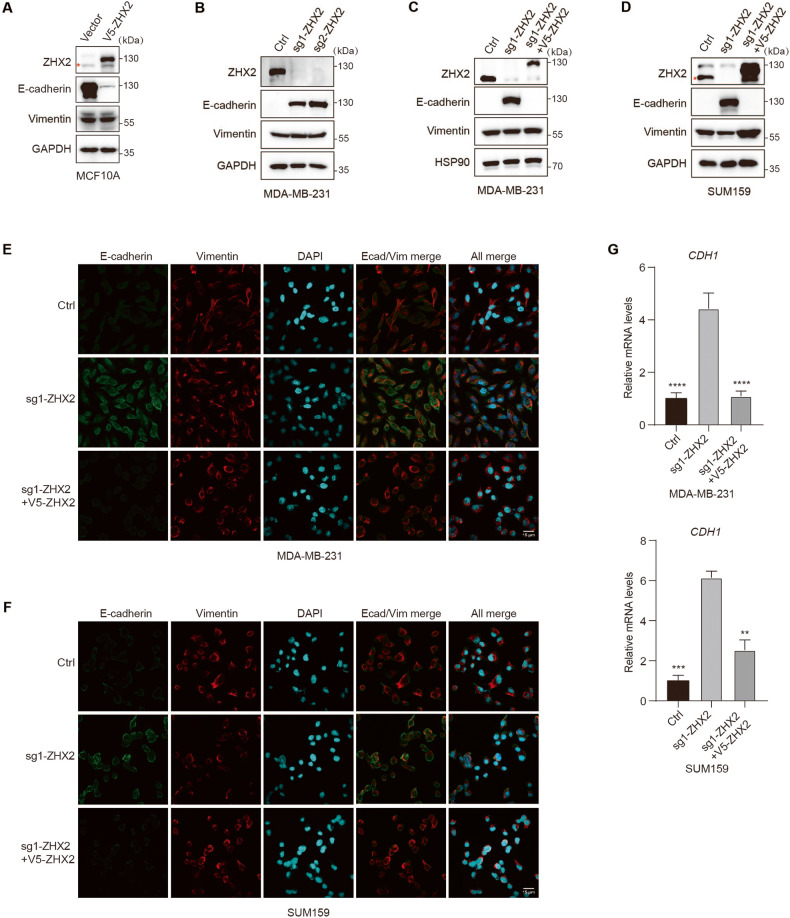
ZHX2 represses E-cadherin expression in TNBC. **A** MCF10A cells infected with lentivirus encoding control vector or V5-ZHX2. Cells were harvested and immunoblotted with antibodies as indicated. **B** MDA-MB-231 cells infected with lentivirus encoding two individual ZHX2 sgRNAs (1 and 2) or control sgRNA. Cells were harvested and immunoblotted with antibodies as indicated. **C**, **D** MDA-MB-231 **C** and SUM159 **D** cells infected with lentivirus encoding sg1-ZHX2 or control sgRNA, and rescued by ectopic expression of V5-ZHX2 in the knockout clones. Expression of epithelial cell marker (E-cadherin) and mesenchymal cell markers (Vimentin) were examined by immunoblotting with antibodies as indicated. * indicates the predicted position. **E**, **F** Immunofluorescence staining of E-cadherin (green) and Vimentin (red) in MDA-MB-231 **E** and SUM159 **F** cells. Scale bar 15 μm. Staining of DAPI (blue) indicates nuclei. **G** qRT-PCR analysis of CDH1 from MDA-MB-231 (upper) and SUM159 (lower) cells. Error bars represent mean ± standard deviation (SD), one-way ANOVA. **p < 0.01; ***p < 0.001; ****p < 0.0001.

To test whether ZHX2 regulates E-cadherin expression, we stably expressed ZHX2 in epithelial MCF10A cells. The result showed that ectopic expression of ZHX2 significantly inhibited protein levels of E-cadherin but not Vimentin compared to control cells (Fig. 2A). Conversely, ZHX2-depleted mesenchymal MDA-MB-231 cells significantly presented abundant protein levels of E-cadherin compared to control cells whereas Vimentin remained unchanged (Fig. 2B). Moreover, we found that E-cadherin increased in both ZHX2-depleted MDA-MB-231 and SUM159 cells could be completely reversed by restoration of ZHX2, suggesting that ZHX2 specifically regulated the expression of E-cadherin. (Fig. 2C, D). Consistent with Western blot results, Immunofluorescence (IF) analysis provided additional evidence for ZHX2 depletion resulting in an increase in E-cadherin in TNBC cells (Fig. 2E, F). Furthermore, we found that the mRNA levels of E-cadherin increased in both ZHX2-depleted MDA-MB-231 and SUM159 cells, but decreased by re-expression of ZHX2 in the deficient cells (Fig. 2G), indicating that ZHX2 played a key role in regulating the transcription of E-cadherin.

### ZHX2 binds to the CDH1 promoter and represses its transcription

**Fig. 3:**
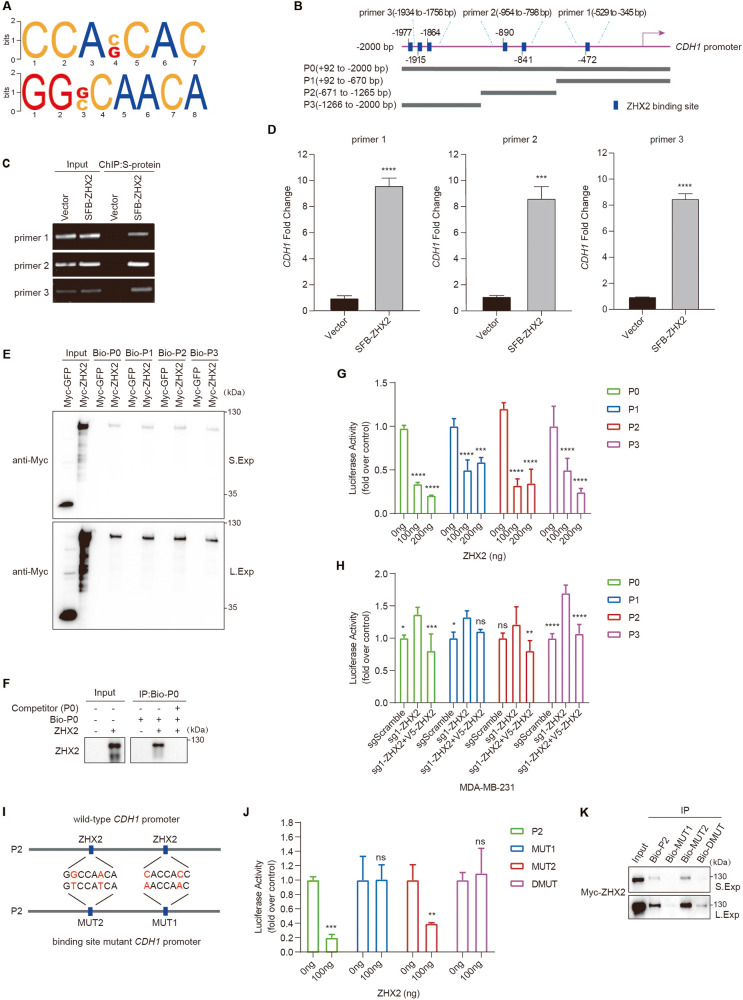
ZHX2 binds to and represses activity of the CDH1 promoter. **A** The predicted binding motif of ZHX2 in genome. **B** Gene sequence analysis was performed to predict positions of putative ZHX2 binding sites in −2 kb human CDH1 promoter (P0, +92 to -2000 bp), accordingly, human CDH1 promoter was divided into three fragments, namely P1 ( + 92 to −670 bp), P2 (−671 to −1265 bp) and P3 (−1266 to −2000 bp), and the primers were designed accordingly (primer 1 (−529 to −345 bp), primer 2 (−954 to −798 bp), primer 3 (−1934 to −1756 bp)) for ChIP assay. **C**, **D** ChIP analysis of the CDH1 promoter was performed in vector or SFB-ZHX2 expressing HEK293T cells. **C** PCR amplification and **D** qRT-PCR of S-protein immunoprecipitated DNA showed enrichment of occupancy of ZHX2 at the −529 to −345 bp, −954 to −798 bp, and −1934 to −1756 bp regions of *CDH1*, which containing the putative ZHX2-binding sequence. Error bars represent mean ± standard deviation (SD), unpaired t-test. ***p < 0.001; ****p < 0.0001. **E** Extracts of transfected HEK293T cells were incubated with streptavidin-agarose beads and biotinylated double-stranded oligonucleotides (Bio-P0-3). Whole extracts and precipitated complexes were subjected to immunoblotting using anti-Myc antibody. P0 and P1-3 truncations with putative ZHX2 binding sites were described as in **B**, S.Exp or L.Exp mean short or long exposure. **F** ZHX2 protein obtained by eukaryotic in vitro translation was incubated with streptavidin-agarose beads and biotinylated double-stranded oligonucleotides (Bio-P0) or competitor (P0). Precipitated complexes were subjected to immunoblotting using anti-ZHX2 antibody. **G** Different amounts of ZHX2 expression vector as indicated were transfected into ZHX2 deficient SUM159 cells together with the P0 or P1-3 CDH1 promoters. Protein lysates were prepared at 48 h following transfection and then used to measure dual luciferase enzyme activity. **H** The full-length P0 or P1-3 CDH1 promoters were transfected in MDA-MB-231 cells as indicated. Luciferase activities were measured as in **G**. **I** Mutations generated in P2 (-671 to -1265 bp) of the human CDH1 promoter. MUT1 carries the mutation CACCACC → AACCAAC, whereas MUT2 carries the mutation GGCCAACA → GTCCATCA. DMUT carries both mutations. **J** ZHX2 expression vector was transfected in SUM159 cells together with wild-type (P2) or mutant CDH1 promoters. Luciferase activities were measured as in **G**. MUT1, MUT2 and DMUT CDH1 promoter oligonucleotides with mutations in putative ZHX2 binding sites were described as in **I**. **K** Extracts of transfected HEK293T cells were incubated with streptavidin-agarose beads and biotinylated double-stranded oligonucleotides. Whole extracts and precipitated complexes were subjected to immunoblotting using anti-Myc antibody. In **G**, **H** and **J**, error bars represent mean ± standard deviation (SD), one-way ANOVA. *p < 0.05; **p < 0.01; ***p < 0.001; ****p < 0.0001; *ns* not significant.

ZHX2 has been reported to bind at CCACCAC, CCAGCAC, GGGCAACA and GGCCAACA DNA sequences (Fig. 3A) [27]. Interestingly, multiple putative ZHX2 binding sites are predicted in the -2 kilobase pair (kb) human CDH1 promoter (in regions -472 base pairs (bp), -841 bp, -890 bp, -1864 bp, -1915 bp, and -1977 bp) (Fig. 3B). To clarify whether ZHX2 binds to CDH1 promoter, ChIP assays were performed and the data showed that ZHX2 bound to the endogenous CDH1 promoter in HEK293T cells (Fig. 3C, D) and SUM159 cells (Supplementary Figure S2A). To further validate this finding, we performed DNA-mediated pull-down assay with biotinylated double-stranded oligonucleotides synthesized based on the CDH1 promoter sequences described in Fig. 3B. As expected, we found that ZHX2 could bind to all CDH1 promoter truncations with putative ZHX2 binding sites (Fig. 3E). Moreover, we used ZHX2 protein obtained by eukaryotic in vitro translation in DNA-mediated pull-down assay. Our results showed that ZHX2 can directly bind to the CDH1 promoter (Fig. 3F). Consistently, when we constructed all CDH1 promoter truncations into pGL4 vector and checked the effect of ZHX2 on the luciferase reporters, the results showed that ZHX2 expression significantly inhibited the transcriptional level of CDH1 (Fig. 3G). Furthermore, we transiently transfected reporter plasmids driven by CDH1 promoter truncations or the CDH1 3’ UTR in ZHX2 deficient cells with or without restoration of ZHX2. The results showed that loss of ZHX2 enhanced the transcriptional activity of CDH1 promoter, which could be completely reversed by overexpression of ZHX2, especially for the full-length promoter P0 and the promoter truncation P3 (Fig. 3H). However, the luciferase activity of the CDH1 3’ UTR reporter gene in either MDA-MB-231 or SUM159 cells was not regulated by ZHX2 (Supplementary Figure S2B). These results indicated that ZHX2 directly bound to CDH1 promoter and repressed its transcription.

To determine the motif of ZHX2 binding to the CDH1 promoter, we generated mutations at the human CDH1 promoter P2 (-671bp to −1265bp). Mutant 1 (MUT1) carries the mutation CACCACC → AACCAAC, MUT2 carries the mutation GGCCAACA → GTCCATCA, and the double mutant (DMUT) carries both indicated mutations (Fig. 3I). When cotransfection was performed with ZHX2 cDNA and the different mutated CDH1 promoter constructs, we found that only the activities of MUT1 and DMUT of CDH1 promoter were not affected by ZHX2 overexpression (Fig. 3J). Hence, we conclude that ZHX2 repressed the transcriptional activity of the CDH1 promoter, likely through binding CCACCAC DNA sequences. To further confirm the findings, we performed a DNA-mediated pull-down assay. As shown, wild-type or MUT2 CDH1 promoter constructs could efficiently precipitate ZHX2 from cells, whereas mutation of the CCACCAC motif in both MUT1 and DMUT resulted in the loss of ZHX2 binding (Fig. 3K). Thus, these results demonstrated that the CDH1 gene was a direct transcriptional target of ZHX2 in triple-negative breast cancer.

### Loss of ZHX2 abrogates migration and invasion of TNBC cells through E-cadherin

**Fig. 4:**
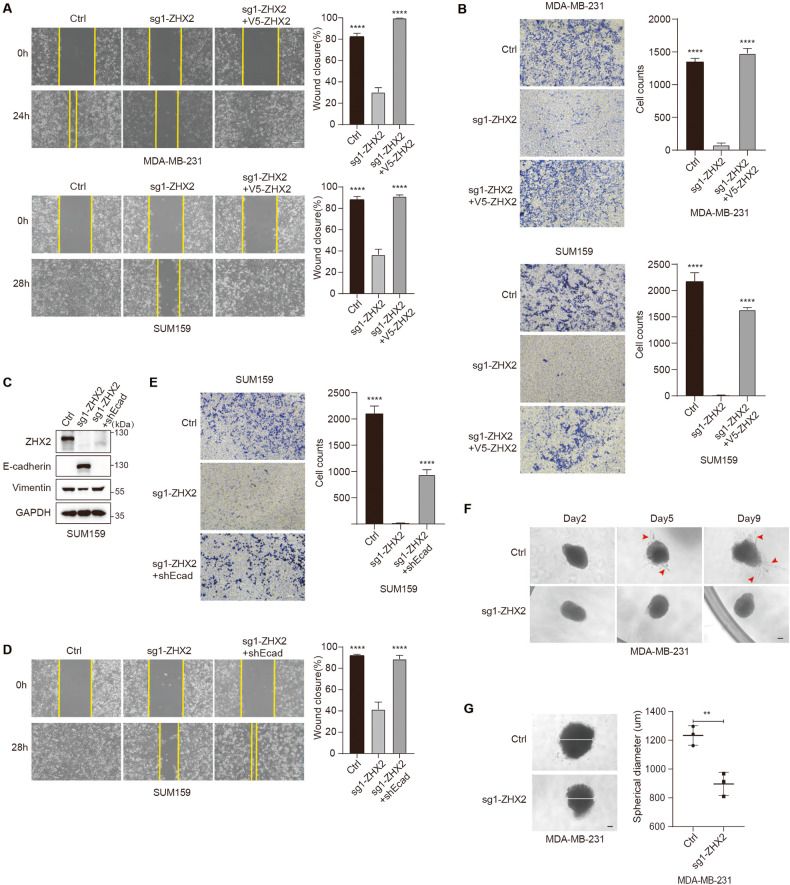
Loss of ZHX2 abrogates migration and invasion of TNBC cells through E-cadherin. **A** Representative scratch-wound images showing the healing ability in indicated MDA-MB-231 (upper) and SUM159 (lower) cells. Scale bar 100 μm. Error bars represent mean ± standard deviation (SD), one-way ANOVA. ****p < 0.0001. **B** The migration ability of MDA-MB-231 (upper) or SUM159 (lower) cells was determined by Transwell migration assay. Scale bar 100 μm. Error bars represent mean ± standard deviation (SD), one-way ANOVA. ****p < 0.0001. **C** Immunoblots from SUM159 cells infected with lentivirus encoding sg1-ZHX2 or control sgRNA, and then followed by knocking down E-cadherin. The expression of E-cadherin and Vimentin were examined by immunoblotting with antibodies as indicated. **D** Representative scratch-wound images showing the healing ability of indicated SUM159 cells. Scale bar 100 μm. Error bars represent mean ± standard deviation (SD), one-way ANOVA. ****p < 0.0001. **E** The migration ability of SUM159 cells, as described in **C**, was determined by Transwell migration assay. Scale bar 100 μm. Error bars represent mean ± standard deviation (SD), one-way ANOVA. ****p < 0.0001. **F** Phase contrast images of ZHX2 control and knock-out MDA-MB-231 organoids grown in 5 μg/ml Collagen I (2, 5, 9 days). The red arrow indicates branching and stellate structures. Scale bar 20 μm. **G** Representative phase contrast images of ZHX2 control and knockout MDA-MB-231 organoids. Quantitative analysis of spherical diameter is shown in the bar graphs. The white line indicates the spherical diameter. Scale bar 20 μm. Error bars represent mean ± standard deviation (SD), unpaired t-test. **p < 0.01.

As E-cadherin is considered a suppressor of tumor metastasis [5], we ask whether low ZHX2 levels can switch mesenchymal cells to a lower metastatic phenotype. Wound healing assays showed that loss of ZHX2 in MDA-MB-231 and SUM159 cells healed the wound much slower than the control cells and the ZHX2 expression restored cells (Fig. 4A and Supplementary Figure S3A-E) while the cell proliferation was not affected (Supplementary Figure S3F, G). Transwell migration assay showed that depletion of ZHX2 decreased the migration of MDA-MB-231 and SUM159 cell lines (Fig. 4B). To address whether E-cadherin could reverse ZHX2 depletion-mediated inhibition of TNBC migration, we knocked down E-cadherin in ZHX2 deficient cell lines (Fig. 4C). Consistently, depletion of ZHX2 markedly inhibited the cell migration while loss of E-cadherin significantly reversed ZHX2 deficiency-mediated suppression of migration of SUM159 cells (Fig. 4D, E). To test the requirement for ZHX2 in the invasion, we cultured control and ZHX2 deficient organoids generated by MDA-MB-231 cells in 3D collagen I. Compared to control organoids, ZHX2 deficient organoids did not form multiple branching and stellate structures that represented the ability of cell invasion (Fig. 4F). In addition, ZHX2 deficient organoids were smaller and less dense than control organoids (Fig. 4G), suggesting reduced invasion and dissemination of cells. Overall, these findings identified that ZHX2 deficiency-caused inhibition of TNBC migration and invasion required its downstream target E-cadherin.

### ZHX2 promotes lung metastasis and correlates with poor survival of TNBC

**Fig. 5:**
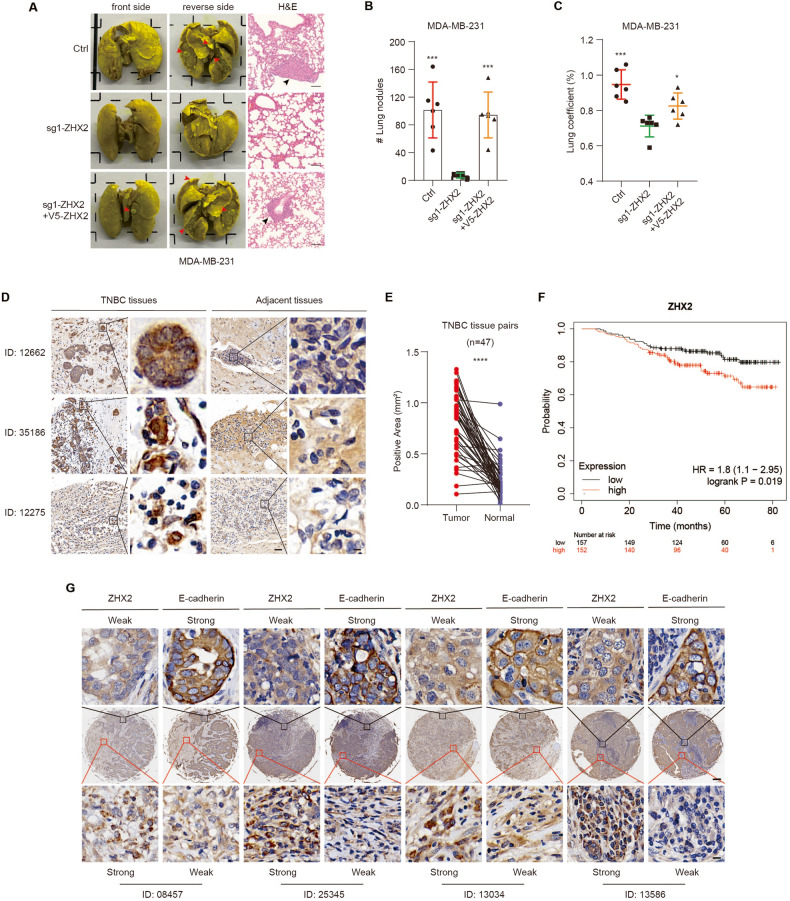
ZHX2 promotes lung metastasis and correlates with poor survival in TNBC. **A** Lung metastasis nodules after intravenous injection of MDA-MB-231 cells infected with lentivirus encoding sg1-ZHX2 or control sgRNA, and rescued by ectopic expression of V5-ZHX2 in the knockout clones. Representative images in (Left and Middle) showed pulmonary surface nodules (red arrows). Representative images in (Right) showed H&E staining for metastatic nodules (black arrows). Scale bar 100 μm. **B** Numbers of lung metastasis nodules of mice injected with the indicated MDA-MB-231 cells. *n* = 6 mice per group, Error bars represent mean ± standard deviation (SD), one-way ANOVA. ***p < 0.001. **C** Quantitative analysis of lung injury in mice in **A**. Lung coefficient = wet lung weight/body weight * 100%. Error bars represent mean ± standard deviation (SD), one-way ANOVA. *p < 0.05; ***p < 0.001. **D**, **E** Representative Immunohistochemistry (IHC) staining images **D** and quantification **E** of ZHX2 protein in TNBC specimens and the corresponding tumor-adjacent tissues. Scale bar 50 μm (left) or 5 μm (right). **F** Survival analysis graphs of breast cancer based on ZHX2 expression. Using a publicly available database (KM plotter; www.kmplot.com), relapse-free survival curves were analyzed by ZHX2 expression. Log-rank P-values were calculated in the KM-plot database. **G** Representative IHC staining images of human TNBC specimens with two staining grades showing the expression correlation between ZHX2 and E-cadherin protein. Scale bar 200 μm (the middle row) or 10 μm (the upper row and lower row).

Previous studies showed that hybrid E/M cells are more metastatic than mesenchymal cells [28]. To further explore the role of ZHX2 in breast cancer lung metastasis, we intravenously injected 5-week-old female athymic nude mice with vector control, ZHX2 deletion or the ZHX2 expression restored MDA-MB-231 cells. ZHX2 depletion in MDA-MB-231 cells significantly reduced the number of visible metastatic nodules on the lung surface of tumor-bearing mice (Fig. 5A, B and Supplementary Figure S4A). Histological analysis confirmed that ZHX2 knockout significantly decreased the number of metastatic foci in the lungs (Fig. 5B). Consistently, the lung coefficient (Lung coefficient = wet lung weight/body weight) of ZHX2 deletion group was significantly lower than that of control group and ZHX2 restoration group, indicating that ZHX2 deletion attenuated lung damage in mice (Fig. 5C). These findings demonstrated a pro-metastatic role of ZHX2 in triple-negative breast cancer and argued that hybrid E/M TNBC cells are less metastatic cells. To validate the clinical relevance of ZHX2 expression, we conducted immunohistochemistry (IHC) staining for ZHX2 on a TNBC tissue microarray (product number: ZL-Brc3N961) containing 48 pairs of TNBC specimens and the corresponding tumor-adjacent tissues. The expression level of ZHX2 in the nucleus of TNBC tissues was significantly higher than that in the adjacent tissues (Fig. 5D, E). The high expression of ZHX2 was also significantly associated with tumor stage (Supplementary Table S2), and poor patient survival (Fig. 5F). Lastly, to further investigate the relevance of CDH1 and ZHX2 in breast cancer patients, we first examined their mRNA levels in breast cancer (*n* = 148) from the gene expression database available through METABRIC [29, 30] (Supplementary Figure S4B). The data showed that the mRNA expression of CDH1 negatively correlated with that of ZHX2 in breast cancer. Consistently, IHC/IF results showed that CDH1 and ZHX2 protein levels were negatively correlated in both mouse (Supplementary Figure S4C) and human primary and metastatic tumors (Fig. 5G and Supplementary Figure S4D). These results demonstrated that ZHX2 level could determine the proportion of hybrid and mesenchymal cells and regulate breast cancer lung metastasis.

## Discussion

**Fig. 6:**
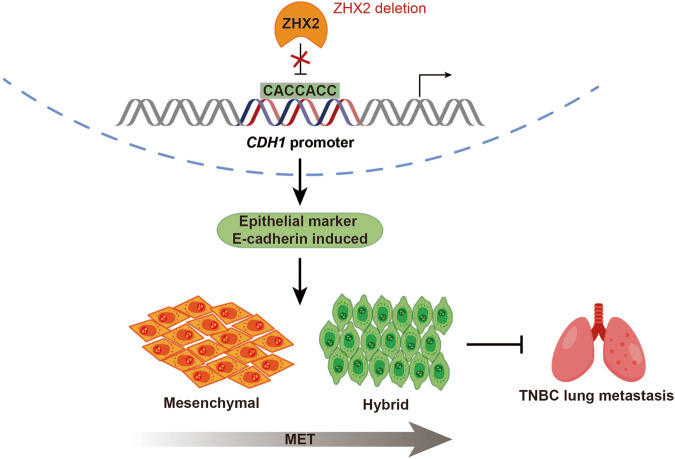
The working model of maintaining the hybrid MET status by ZHX2. The direct binding between ZHX2 and the CDH1 promoter by recognizing the CCACCAC DNA sequence. ZHX2 deficiency upregulated the E-cadherin expression to enrich hybrid MET cells in mesenchymal-like TNBC cells, which further remarkably inhibited the metastasis of TNBC cells.

Results presented in this paper highlighted ZHX2 function in maintaining the hybrid E/M phenotype by regulating the transcription of epithelial marker, E-cadherin, in triple-negative breast cancer (Fig. 6). We described the direct binding between ZHX2 and the CDH1 promoter by recognizing the CCACCAC DNA sequence. ZHX2 deficiency upregulated the E-cadherin expression to enrich hybrid MET cells in mesenchymal-like TNBC cells, which further remarkably inhibited the metastasis of TNBC cells. Notably, ZHX2 expressions were amplified in diverse areas of the tumor mass and negatively correlated with E-cadherin expressions in patients with TNBC, which is associated with poor TNBC prognosis.

Recent studies have shown that EMT occurs in a progressive manner, characterized by cells that express varying levels of epithelial and mesenchymal markers and exhibit intermediate morphological, transcriptional, and epigenetic signatures between epithelial and mesenchymal cell status [9, 31–35]. The intermediate state between the epithelial and fully mesenchymal states is referred to as the partial, incomplete or hybrid EMT state [28]. Plasticity of hybrid state allows cancer cells to adapt to environmental stress during malignancy progress, which highlights the importance of the status of hybrid E/M. However, the knowledge about hybrid E/M is relatively limited compared to the extensive researches on the EMT. In this study, we have demonstrated that ZHX2 deficiency reactivated the E-cadherin transcription and generated more hybrid MET cells in mesenchymal-like TNBC cells, and pointed that those cells lost their original metastatic and invasive capacity compared with fully mesenchymal cells. In addition, our findings were consistent with previous studies showing that E-cadherin suppresses cell invasion and acts as a metastasis suppressor [36, 37].

Hybrid E/M phenotype has been detected in CTCs in the blood of human patients with non-small cell lung cancer, gastric cancer, liver cancer, colorectal cancer, nasopharyngeal carcinoma and prostate cancer [38–43]. Previous studies used three markers as a tool to isolate and characterize six distinctive cell subpopulations, and divide them into five EMT states (epithelial tumor cells, early hybrid EMT state, hybrid EMT state, late hybrid EMT state, and mesenchymal tumor cells) [28, 34]. The studies also found that early hybrid EMT state and hybrid EMT state had the strongest metastatic ability, while late hybrid EMT state and mesenchymal tumor cells had strong invasive potential. Hybrid E/M state in our model was characterized by low metastatic and invasive capacity compared with mesenchymal tumor cells. Although there was no difference in their proliferative capacity in 2D culture (Supplementary Figure S3F, G), hybrid E/M showed a markedly low proliferative capacity in 3D culture (Fig. 4G). Previous study argued that the most novel therapeutic concept is to fix cells at a given location on the epithelial-mesenchymal axis to prevent entry into the range of states that may be required to promote different stages of the metastatic cascade. Our study may contribute to the development of targeted therapeutic strategies for these highly plastic and dynamic cell populations, and provide a way to enhance the personalization of cancer treatment.

Intermediate aspects of EMT are controlled by distinct transcriptional and signaling processes. For example, mathematical modeling suggested that OVOL2 restricts EMT and induces MET by directly inhibiting ZEB1 [33, 44–46]. MiR-200 family miRNAs repress the expression of ZEB1 and ZEB2, thereby preserving the epithelial phenotype of cancer cells [47–49]. Likewise, ΔNp63 promotes a hybrid EMT state in basal like breast cancer by simultaneously increasing Slug and Axl expression, while also increasing the expression of miR-205 to silence ZEB1/2 for preventing loss of epithelial characteristics [50, 51]. Recently, FAT1 deletion has been reported to promote a hybrid EMT state in mouse models of skin squamous cell carcinoma and lung tumors. The mechanism by which FAT1 deletion promotes a hybrid EMT state is that loss of function of FAT1 promotes ZEB1 expression that stimulates the mesenchymal state [52]. However, previous result showed that knockdown of ZEB1 in TNBC genetically engineered mouse model organoids decreased distinct sets of mesenchymal genes without effect on epithelial gene expression such as E-cadherin [26]. Consistently, our study showed that deficiency of ZEB1 could not reactivate E-cadherin expression to maintain the hybrid MET phenotype in TNBC cells (Supplementary Figure S1B, C). It has been proposed that the transition between epithelial and mesenchymal phenotypes is determined by the levels of ZEB1 and SIP1, whereby once a critical threshold level for miR-200 promoter repression (or relief of repression) is reached, induction of the double-negative feedback loop is precipitated, leading to cellular reprogramming and generation of a bistable state [53]. Further research found that knockdown of both ZEB1 and ZEB2, but not single knockdown of one or the other, was required to induce E-cadherin expression and an MET like morphological change, indicating a redundancy in their function [47]. In addition, the switch from Prrx1b to Prrx1a governs EMT plasticity in pancreatic ductal adenocarcinoma. While Prrx1a overexpression is associated with increased E-cadherin expression, decreased invasion and MET, Prrx1b overexpression decreases E-cadherin expression, increases invasion and promotes EMT [54]. Despite important advances in our understanding of the mechanisms by which different transcription factors can induce EMT or MET, specific regulatory elements and mechanism that stabilize the hybrid MET phenotype in cancer cells, or facilitate the transition from a hybrid state to the full EMT or MET remain poorly understood. Our study not only identified that ZHX2 is a direct transcriptional repressor of E-cadherin, but also suggested that loss of ZHX2 enriched hybrid MET TNBC cells by boosting E-cadherin expression. Notably, ZHX2 specifically regulates MET progression by directly regulating the epithelial marker expression, rather than through synergizing with other EMT-TFs.

Since identifying different E/M states may predict the metastatic potential, future studies will be required to assess the existence and involvement of the mechanisms by which ZHX2 regulates E-cadherin expression in different TNBC molecular subtypes and other cancer types. Moreover, based on the expression of ZHX2, clinicians may develop more effective, personalized approaches for cancer treatment.

## Supplementary information

ZHX2 deficiency enriches hybrid MET cells through regulating E-cadherin expression

Original Data File

checklist

Table S1

Table S2

Table S3

Table S4

## Data Availability

The datasets and materials used and/or analyzed during the current study are included in this published article and its Supplementary Information files. Additional data are available from the corresponding author on reasonable request.
