# Supplementary material for: ZHX2 deficiency enriches hybrid MET cells through regulating E-cadherin expression
Source: Cell Death Dis. 2023 Jul 17;14(7):444. doi: 10.1038/s41419-023-05974-y (PMC10352340; doi:10.1038/s41419-023-05974-y)
Supplement: Supplementary file 1 — ZHX2 deficiency enriches hybrid MET cells through regulating E-cadherin expression [file 41419_2023_5974_MOESM1_ESM.docx]

SUPPLEMENTARY INFORMATION

ZHX2 deficiency enriches hybrid MET cells through regulating E-cadherin expression

Yan He^1, 5^, Qimin Zhang^1, 5^, Yuanhong Chen^1^, Yingjian Wu^1^, Yuan Quan^2*^, Weihua Chen^1^, Jing Yao^3, 4^, Peijing Zhang^1*^

^5^These authors contributed equally: Yan He, Qimin Zhang

*Correspondence: [yuanquan1011@gmail.com](mailto:yuanquan1011@gmail.com); zhangpeijing@hust.edu.cn

**Keywords:** Triple-negative breast cancer; migration; dissemination; ZHX2; E-cadherin; hybrid MET

**Figure S1**


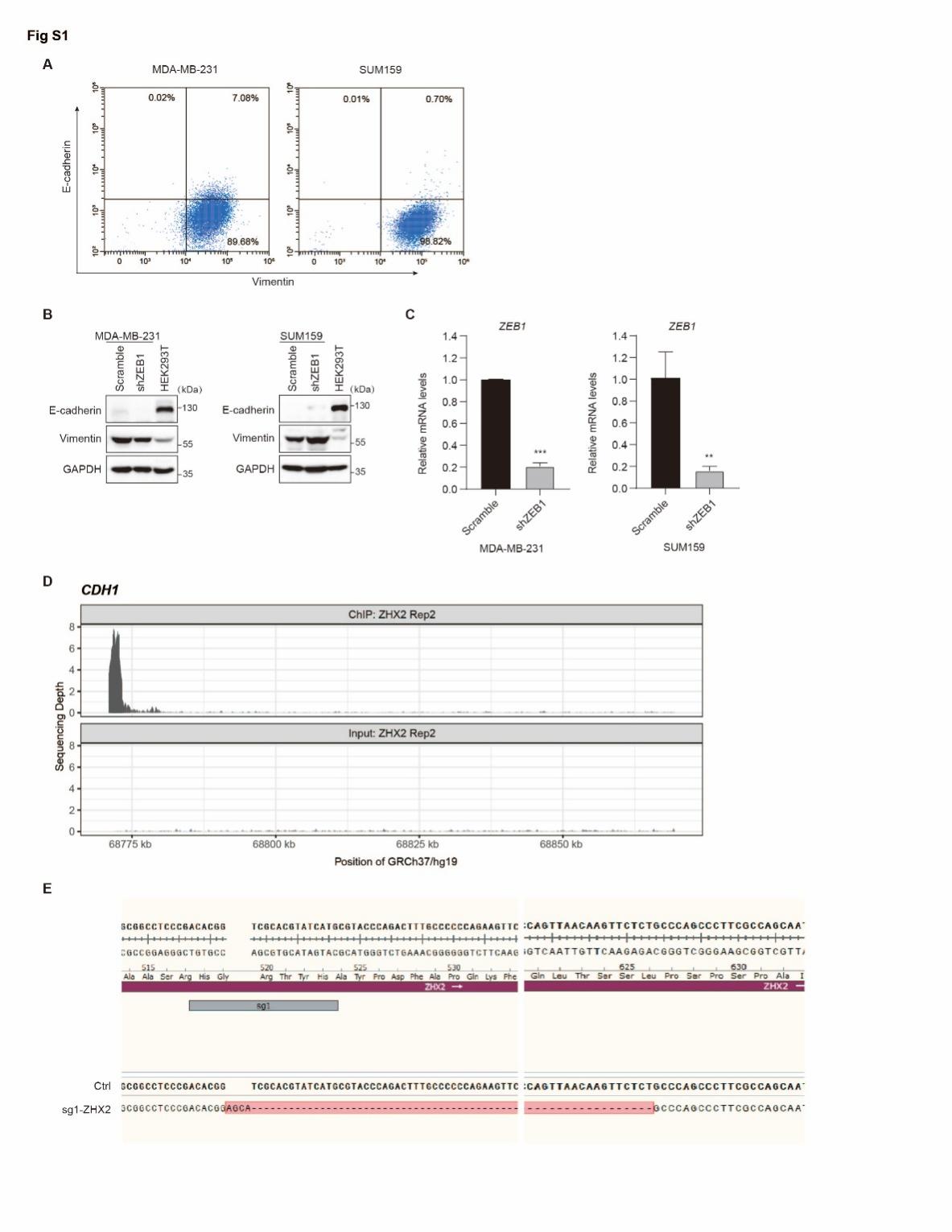


**Fig. S1 Amount of hybrid E/M cells are present in TNBC cells**(A) Flow cytometry analysis of epithelial marker E-cadherin and mesenchymal marker Vimentin profiles in MDA-MB-231 and SUM159 cells. (B) Immunoblot from MDA-MB-231 (left) and SUM159 (right) cells infected with lentivirus encoding ZEB1 shRNA or control shRNA (Scramble). HEK293T cells are used as E-cadherin expression positive control. (C) Real-time quantitative PCR (RT-qPCR) for ZEB1 in MDA-MB-231 (left) and SUM159 (right) cells. Error bars represent mean ± standard deviation (SD), unpaired t-test. **p < 0.01; ***p < 0.001. (D) UCSC Genome Browser snapshots of CDH1 loci with ChIP-seq enrichment on ZHX2. Data are scaled to be proportional to aligned read depth. (E) ZHX2 was knocked out by CRISPR-Cas9 system in MDA-MB-231 cells. Genomic DNA was purified and the targeted locus was amplified by PCR. Representative Sanger sequencing was used to confirm the ZHX2 knock out efficiency.

**Figure S2**


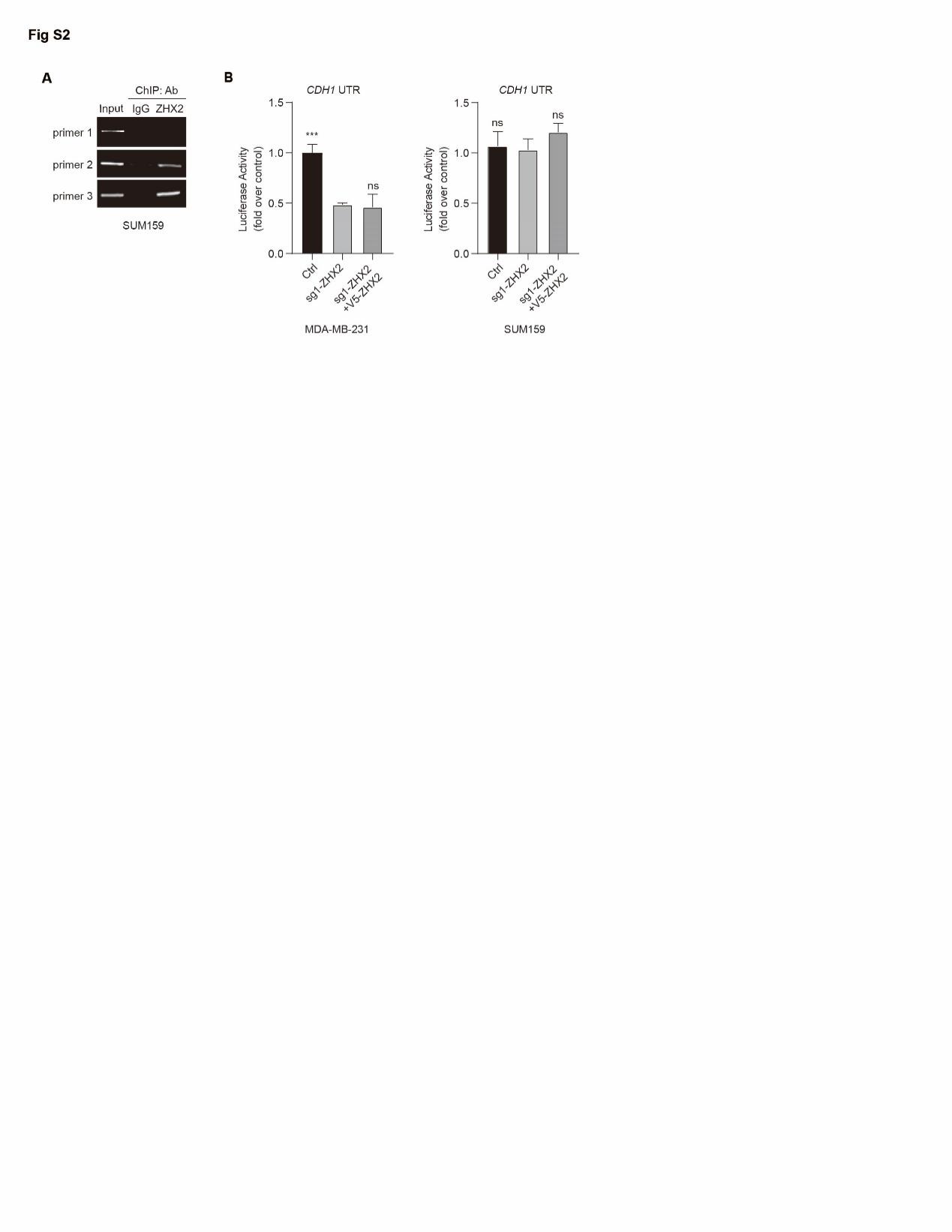


**Fig. S2 ZHX2 transcriptionally regulates E-cadherin through promoter occupancy.**(A) ChIP analysis of the CDH1 promoter in SUM159 cells using ZHX2 antibody or isotype IgG control. PCR amplification showed enrichment of occupancy of ZHX2 at the −954 to −798 bp and −1934 to −1756 bp regions of E-cadherin, which containing the putative ZHX2-binding sequence. There was no enrichment at the −529 bp to −345 bp region. (B) Luciferase activity of the E-cadherin 3′UTR reporter gene in indicated MDA-MB-231 (left) and SUM159 (right) cells.

**Figure S3**


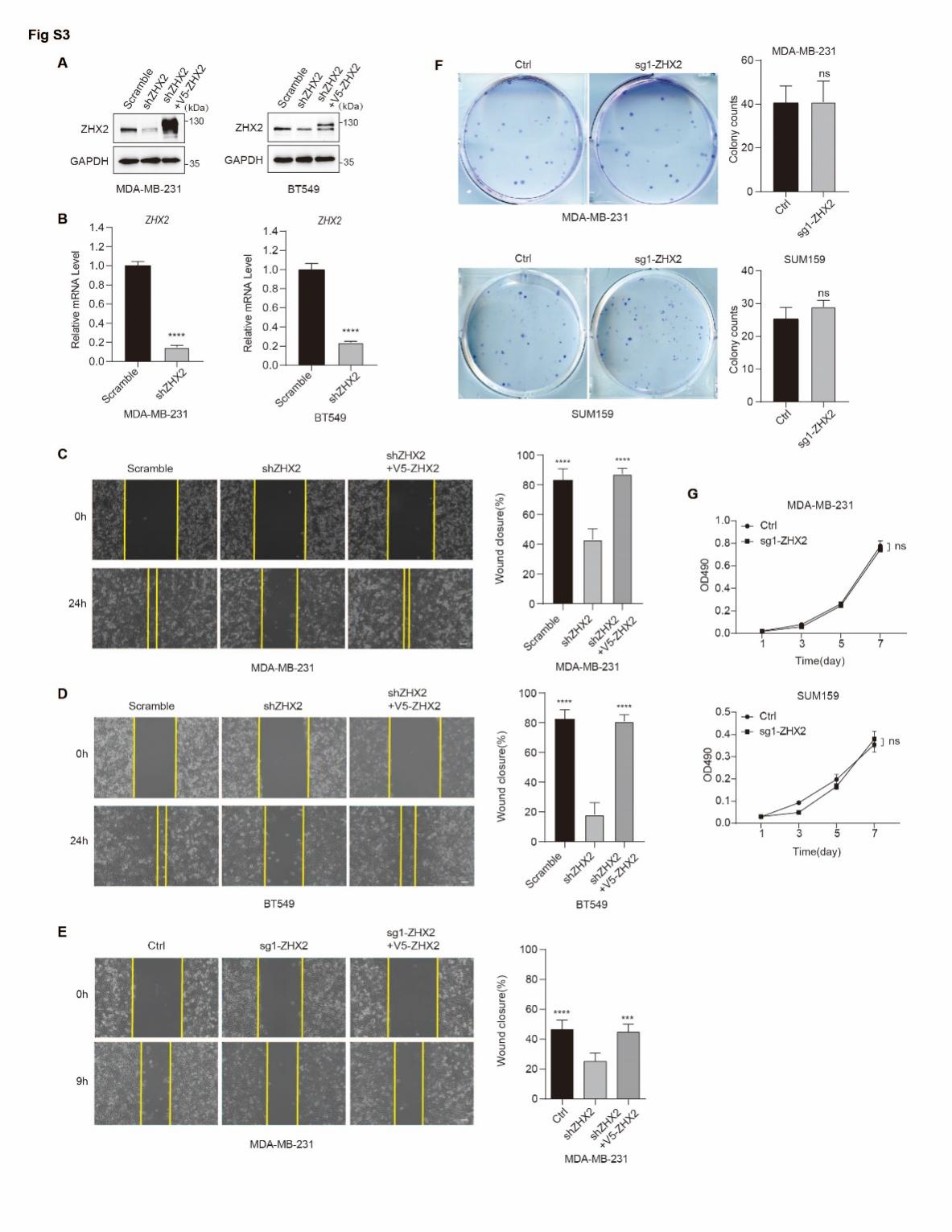


**Fig. S3 ZHX2 is essential for triple-negative breast cancer cell migration, but not proliferation.** (A–D) Immunoblot of cell lysates (A), RT-qPCR (B), Wound Healing assays (C, D) of MDA-MB-231 or BT549 cells infected with lentivirus encoding the ZHX2 shRNA or control shRNA (Scramble), and rescued by ectopic expression of V5-ZHX2 in the knock-down clones. Error bars represent mean ± standard error of the mean (SEM), unpaired t-test, one-way ANOVA. ****p < 0.0001. (E) Representative scratch-wound images showing the healing ability in indicated MDA-MB-231 cells. Scale bar 100 μm. Error bars represent mean ± standard deviation (SD), one-way ANOVA. ***p < 0.001; ****p < 0.0001. (F, G) 2D colony formation assays (F), cell proliferation assays (G) of MDA-MB-231 or SUM159 cells infected with encoding sg1-ZHX2 or control sgRNA (Ctrl). Error bars represent mean ± standard error of the mean (SEM), unpaired t-test. ns, not significant.

**Figure S4**


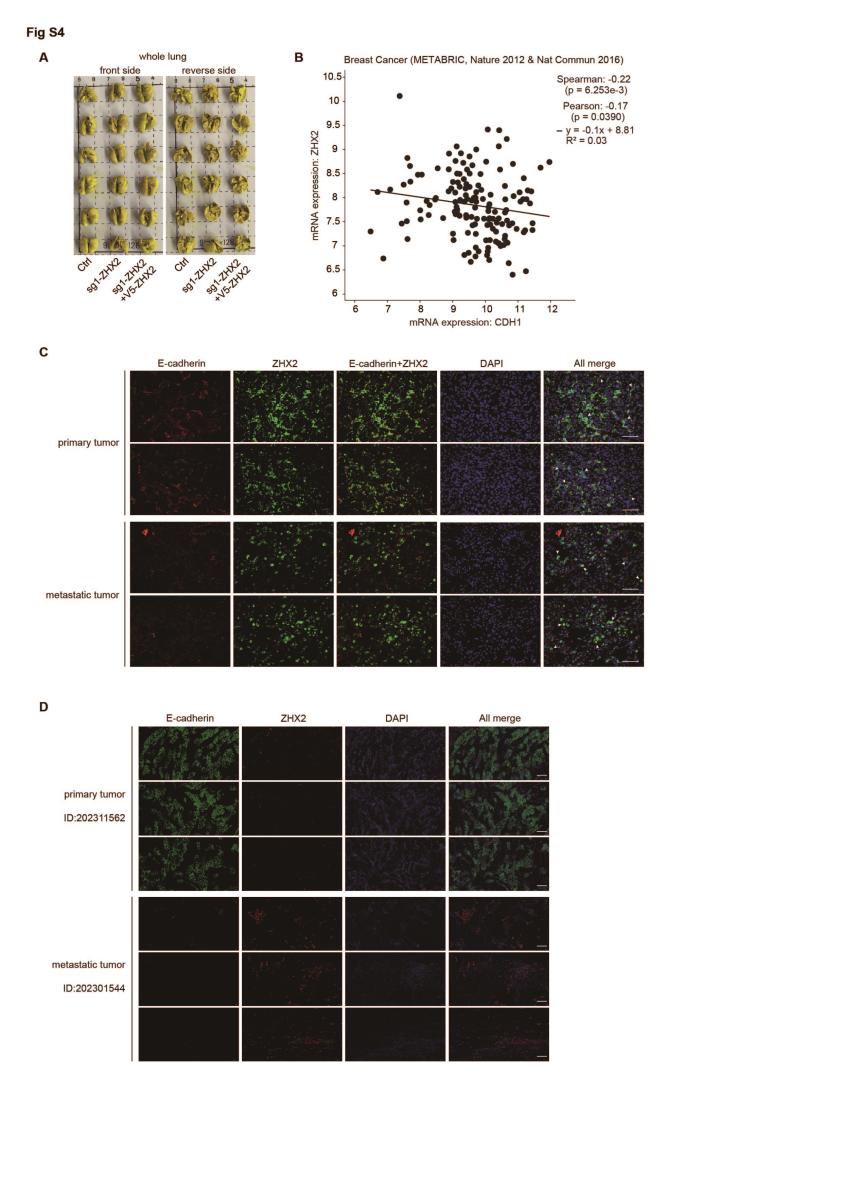


**Fig. S4 CDH1 negatively correlated with ZHX2 in breast cancer.** (A) Shown are pulmonary surface nodules images. Lung metastasis nodules after intravenous injection of MDA-MB-231 cells infected with lentivirus encoding sg1-ZHX2 or control sgRNA (Ctrl), and rescued by ectopic expression of V5-ZHX2 in the knock-out clones (n = 6 mice per group). (B) Correlation between ZHX2 and CDH1 mRNA level in breast cancer (n = 148) from the gene expression database available through METABRIC. (C) Immunofluorescence staining of ZHX2 (green) and E-cadherin (red) in paired mouse primary tumors (n = 2) and metastatic tumors (n = 2). Scale bar 100 μm. Staining of DAPI (blue) indicates nuclei. (D) Immunofluorescence staining of E-cadherin (green) and ZHX2 (red) in paired human primary tumors and metastatic tumors. Scale bar 50 μm. Staining of DAPI (blue) indicates nuclei.
