# Supplementary figures and images for: ZHX2 deficiency enriches hybrid MET cells through regulating E-cadherin expression
Source: Cell Death Dis. 2023 Jul 17;14(7):444. doi: 10.1038/s41419-023-05974-y (PMC10352340; doi:10.1038/s41419-023-05974-y)

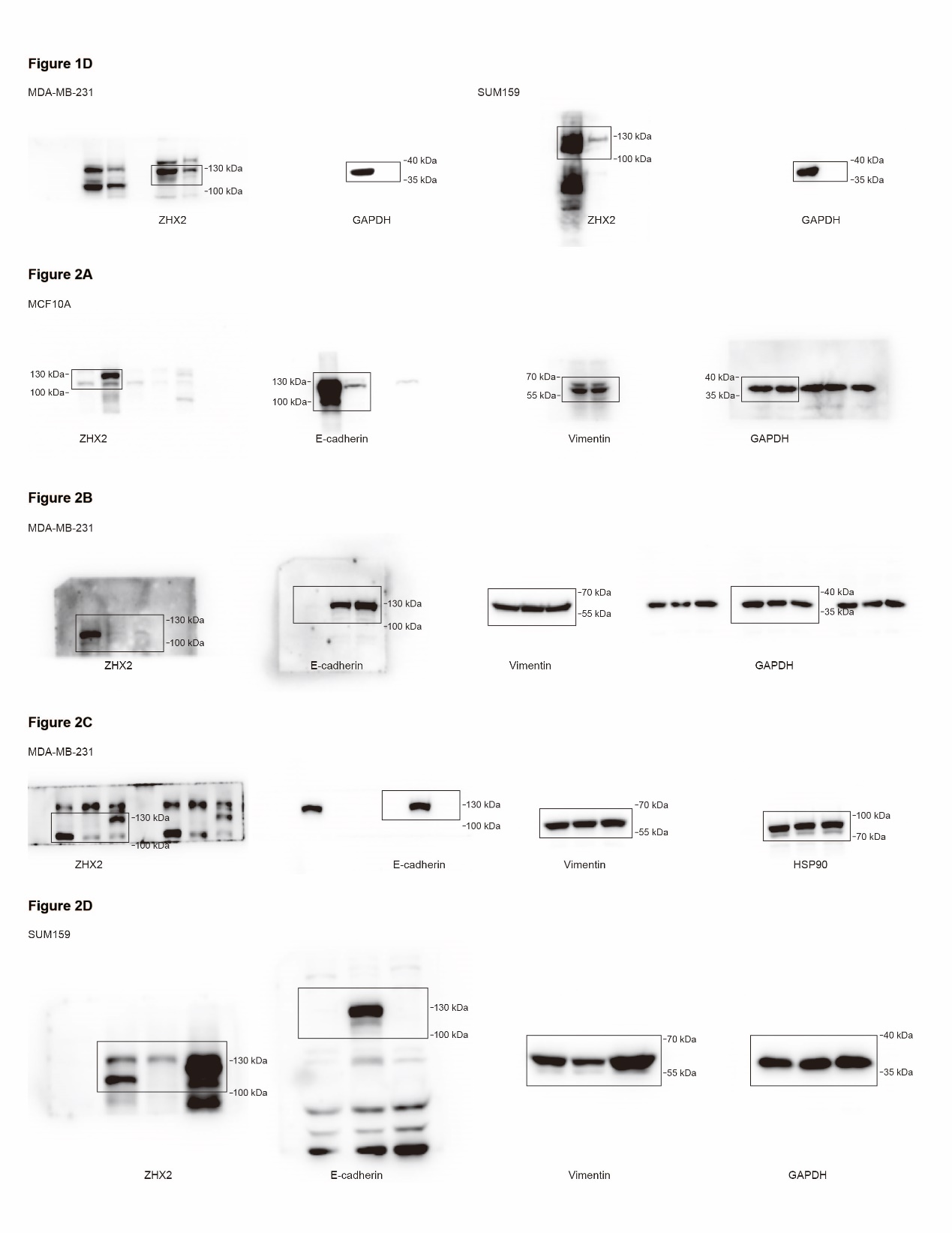


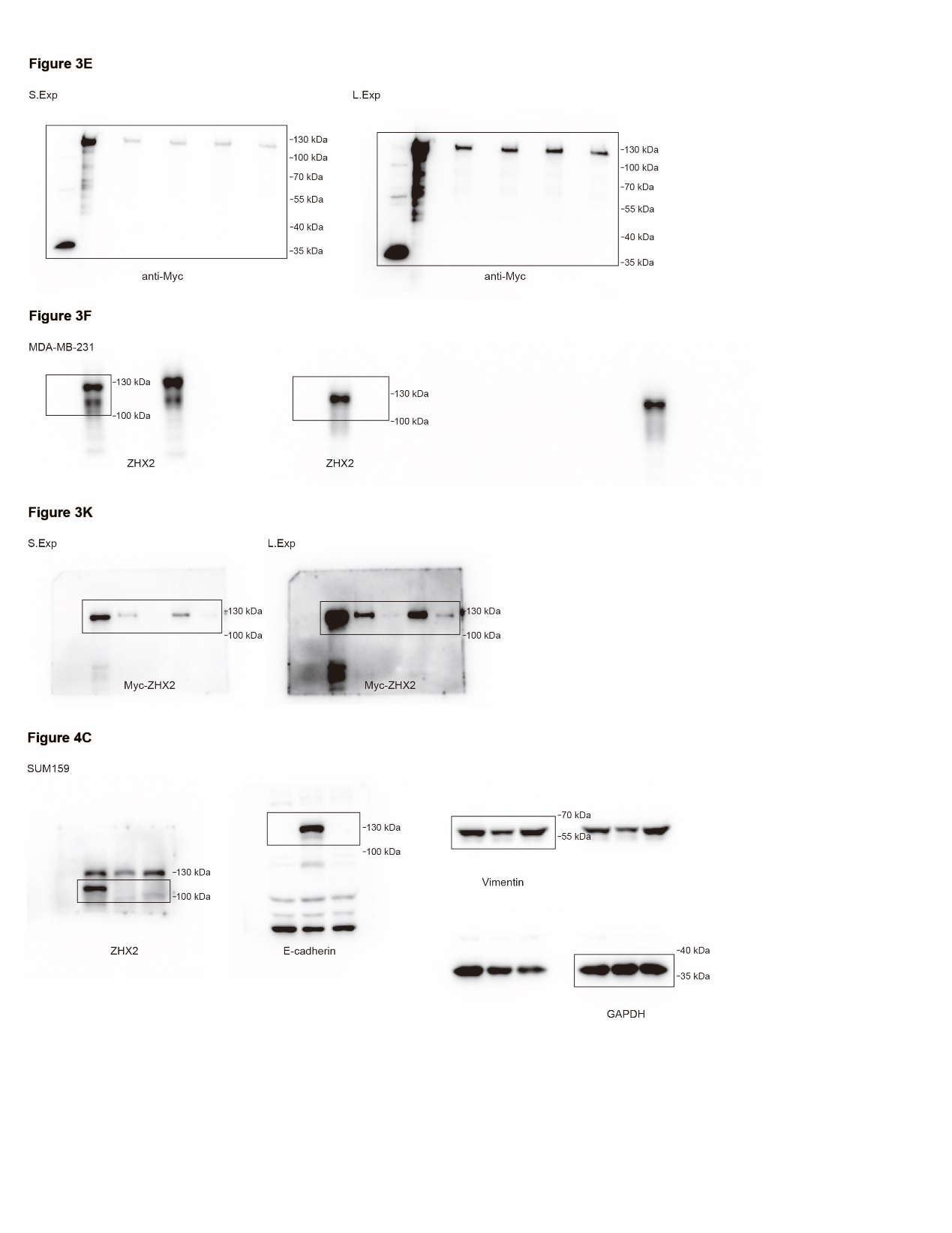


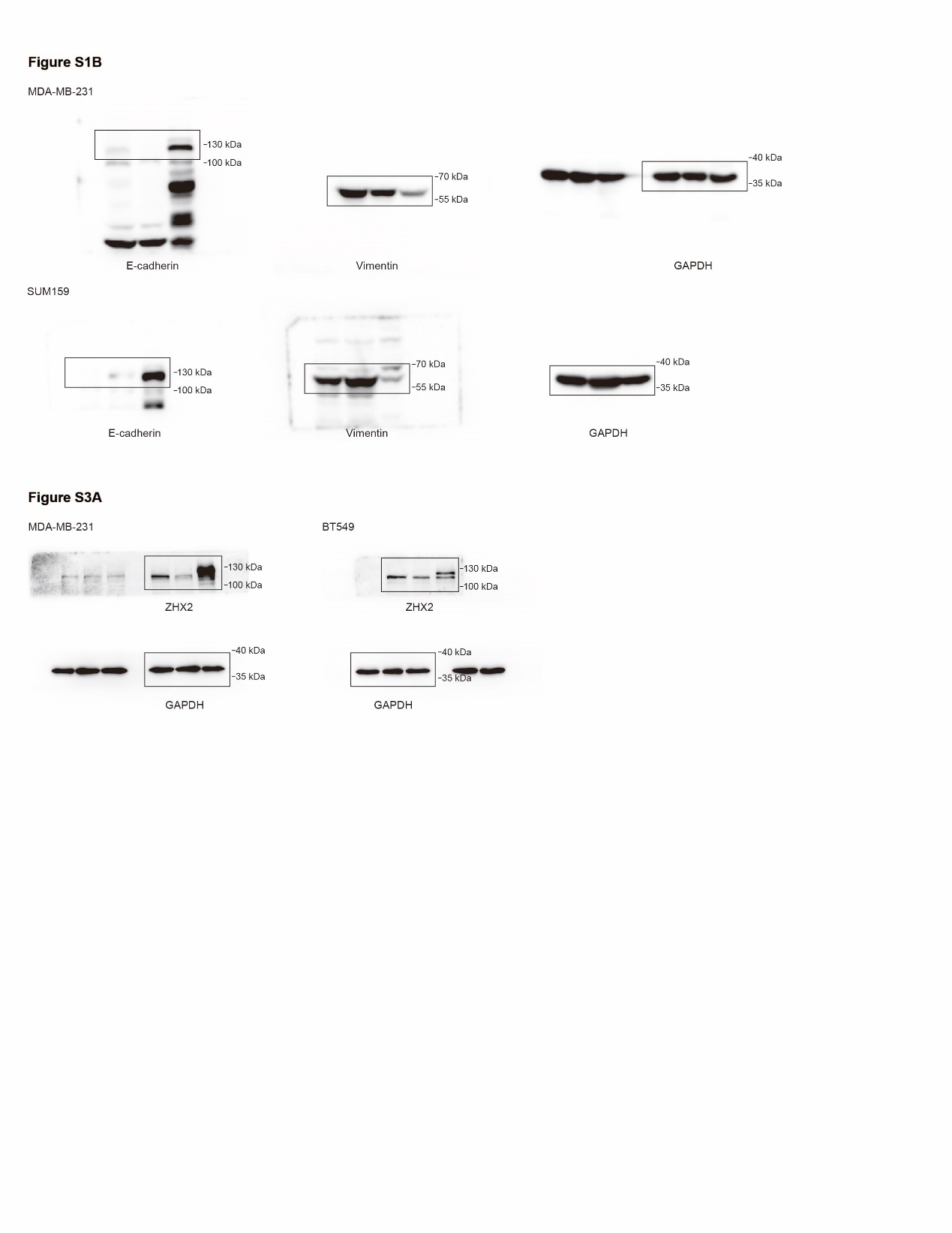

Supplement: Supplementary file 2 — Original Data File [file 41419_2023_5974_MOESM2_ESM.docx]
