# Supplementary material for: ZHX2 deficiency enriches hybrid MET cells through regulating E-cadherin expression
Source: Cell Death Dis. 2023 Jul 17;14(7):444. doi: 10.1038/s41419-023-05974-y (PMC10352340; doi:10.1038/s41419-023-05974-y)
Supplement: Supplementary file 4 — Table S1 [file 41419_2023_5974_MOESM4_ESM.docx]

**Table S1 List of transcription factors precipitated by the CDH1 promoter.**

P51825|AFF1, Q9UHB7|AFF4, P25440|BRD2, Q68CP9|ARID2, Q4LE39|ARI4B, Q99856|ARI3A, P29374|ARI4A, Q8IVW6|ARI3B, Q8NFD5|ARI1B, Q14865|ARI5B, O14497|ARI1A, Q9NZI7|UBIP1, Q12800|TFCP2, O75534|CSDE1, P67809|YBOX1, P16989|YBOX3, P39880|CUX1, Q9UPW6|SATB2, P11474|ERR1, P85037|FOXK1, Q12948|FOXC1, P55316|FOXG1, Q08050|FOXM1, Q9H334|FOXP1, Q8IVH2|FOXP4, P78347|GTF2I, Q92786|PROX1, O95243|MBD4, Q9UIF8|BAZ2B, Q9UIF9|BAZ2A, P51608|MECP2, Q9UIS9|MBD1, Q5SXM2|SNPC4, Q99459|CDC5L, Q99543|DNJC2, Q9P2K3|RCOR3, Q15554|TERF2, Q8IYH5|ZZZ3, Q8TAQ2|SMRC2, Q92922|SMRC1, Q5VVJ2|MYSM1, Q15361|TTF1, Q8N108|MIER1, P28370|SMCA1, O60264|SMCA5, Q6PJG2|MDEAS, Q16656|NRF1, P04637|P53, P14859|PO2F1, P10589|COT1, P49116|NR2C2, P13056|NR2C1, P48380|RFX3, P22670|RFX1, O95644|NFAC1, Q12968|NFAC3, Q14934|NFAC4, O94916|NFAT5, P42224|STAT1, P40763|STAT3, O15119|TBX3, Q8IWI9|MGAP, Q96IZ0|PAWR, O43422|P52K, O95365|ZBT7A, Q8NCP5|ZBT44, O15062|ZBTB5, Q13105|ZBT17, O43167|ZBT24, O43298|ZBT43, Q9HBE1|PATZ1, Q9ULJ3|ZBT21, Q96JB3|HIC2, P52739|ZN131, Q9H116|GZF1, Q96DT7|ZBT10, P10074|TZAP, Q9NUA8|ZBT40, O95625|ZBT11, Q5SVQ8|ZBT41, Q86T24|KAISO, Q96JM7|LMBL3, O94776|MTA2, Q13330|MTA1, Q8WXI9|P66B, Q9BTC8|MTA3, Q86YP4|P66A, O75928|PIAS2, Q8N2W9|PIAS4, O75925|PIAS1, Q12986|NFX1, Q6ZNB6|NFXL1, Q15796|SMAD2, Q13485|SMAD4, Q8NB50|ZFP62, Q9P2J8|ZN624, Q99676|ZN184, Q9UL36|ZN236, O75820|ZN189, Q14590|ZN235, Q9UEG4|ZN629, Q76KX8|ZN534, Q96PQ6|ZN317, Q9NYW8|RBAK, Q96K58|ZN668, Q15776|ZKSC8, Q96MX3|ZNF48, Q5JVG8|ZN506, Q9NTW7|ZF64B, P49711|CTCF, Q969J2|ZKSC4, Q9ULD5|ZN777, P17029|ZKSC1, Q9Y462|ZN711, P52747|ZN143, O60315|ZEB2, Q8N720|ZN655, P37275|ZEB1, Q9UQR1|ZN148, Q9Y2X9|ZN281, Q9H5V7|IKZF5, Q9UI36|DACH1, Q9Y608|LRRF2, Q32MZ4|LRRF1, Q9UBB9|TFP11, Q9Y5B6|PAXB1, P16383|GCFC2, P17480|UBF1, Q06945|SOX4, Q08945|SSRP1, Q969G3|SMCE1, Q8WY36|BBX, Q9UGU5|HMGX4, Q86U86|PB1, Q96NM4|TOX2, O96028|NSD2, O75717|WDHD1, O14686|KMT2D, Q12873|CHD3, Q14839|CHD4, Q86UP3|ZFHX4, Q9UKY1|ZHX1, Q9Y6X8|ZHX2, Q8IX15|HOMEZ, Q9UIU6|SIX4, Q9H2P0|ADNP, Q6IQ32|ADNP2, O75132|ZBED4, O96006|ZBED1, Q49AG3|ZBED5, O15015|ZN646, P0CJ78|ZN865, Q9BWE0|REPI1, Q9H5H4|ZN768, Q6ZN55|ZN574, Q03112|MECOM, Q8N1W2|ZN710, Q9HAZ2|PRD16, P57071|PRD15, P17010|ZFX, Q92766|RREB1, Q6IQ21|ZN770, Q9Y2D9|ZN652, Q66K89|E4F1, Q8TD17|ZN398, Q9NSC2|SALL1, Q9NQV6|PRD10, O15090|ZN536, Q2QGD7|ZXDC, Q9H165|BC11A, Q92618|ZN516, Q9H4Z2|ZN335, O75362|ZN217, O95409|ZIC2, P56270|MAZ, P10071|GLI3, O95785|WIZ, Q13029|PRDM2, Q96T25|ZIC5, Q9Y467|SALL2, P25490|TYY1, P10070|GLI2, Q14119|VEZF1, O60281|ZN292, Q96CK0|ZN653, Q96JP5|ZFP91, Q2TB10|ZN800, Q8N1G0|ZN687, Q13129|RLF, Q96KM6|Z512B, Q02086|SP2, Q63HK5|TSH3, Q96ME7|ZN512, Q6ZN18|AEBP2, Q92610|ZN592, Q9H582|ZN644, Q9UHK0|NUFP1, Q15629|TRAM1, O95251|KAT7, Q92794|KAT6A, P29375|KDM5A, Q9UGL1|KDM5B, Q9Y4F5|C170B, O00327|BMAL1, Q99081|HTF4, O15516|CLOCK, P35869|AHR
