# Supplementary material for: ZHX2 deficiency enriches hybrid MET cells through regulating E-cadherin expression
Source: Cell Death Dis. 2023 Jul 17;14(7):444. doi: 10.1038/s41419-023-05974-y (PMC10352340; doi:10.1038/s41419-023-05974-y)
Supplement: Supplementary file 5 — Table S2 [file 41419_2023_5974_MOESM5_ESM.docx]

**Table S2 ZHX2 expression in breast cancer patients**

| **Clinical Info** | | | **No. of cases** | | **ZHX2 expression** | |  |  |  |  |
| --- | --- | --- | --- | --- | --- | --- | --- | --- | --- | --- |
|  |  |  |  | | **Low** | **High** | **Logrank P** | | **HR** | |
| All patients | |  | 1089 | | 272 | 817 | 0.077 | | 1.41(0.96-2.08) | |
| **Gender** |  |  |  | |  |  |  | |  |  |
| Male |  |  | 12 | |  |  |  | |  |  |
| Female |  |  | 1077 | | 270 | 807 | 0.079 | | 1.41(0.96-2.08) | |
| **Mutation burden** | | |  | |  |  |  | |  |  |
| low |  |  | 485 | | 176 | 309 | 0.36 | | 0.79(0.48-1.3) | |
| high |  |  | 493 | | 221 | 272 | 0.055 | | 1.62(0.98-2.66) | |
| **Pathologic stage** | | |  | |  |  |  | |  |  |
| I |  |  | 180 | | 86 | 94 | 0.14 | | 0.46(0.16-1.32) | |
| II |  |  | 619 | | 163 | 456 | 0.022* | | 2.11(1.1-4.05) | |
| III |  |  | 247 | | 141 | 106 | 0.028* | | 0.48(0.25-0.94) | |
| IV |  |  | 20 |  | 4 | 16 | 0.01* |  | 0.19(0.04-0.79) | |
| *Means significant difference. | | | | |  |  |  |  |  |  |
