# Supplementary material for: ZHX2 deficiency enriches hybrid MET cells through regulating E-cadherin expression
Source: Cell Death Dis. 2023 Jul 17;14(7):444. doi: 10.1038/s41419-023-05974-y (PMC10352340; doi:10.1038/s41419-023-05974-y)
Supplement: Supplementary file 6 — Table S3 [file 41419_2023_5974_MOESM6_ESM.docx]

**Table S3 The sequence of primers**

| ZHX2 | Forward:5′-AAAGTACGACTCCCTATCCGAC-3′ |
| --- | --- |
|  | Reverse:5′-GGTTGGTGGTTTCGATGGACT-3′ |
| CDH1 | Forward:5′-ATTTTTCCCTCGACACCCGAT-3′ |
|  | Reverse:5′-TCCCAGGCGTAGACCAAGA-3′ |
| ZEB1 | Forward:5′-TGCACTGAGTGTGGAAAAGC-3′ |
|  | Reverse:5′-TGGTGATGCTGAAAGAGACG-3′ |
| GAPDH | Forward:5’-GGTGGTCTCCTCTGACTTCAA-3′ |
|  | Reverse:5’-GTTGCTGTAGCCAAATTCGTTGT-3′ |
