# Supplementary material for: ZHX2 deficiency enriches hybrid MET cells through regulating E-cadherin expression
Source: Cell Death Dis. 2023 Jul 17;14(7):444. doi: 10.1038/s41419-023-05974-y (PMC10352340; doi:10.1038/s41419-023-05974-y)
Supplement: Supplementary file 7 — Table S4 [file 41419_2023_5974_MOESM7_ESM.docx]

**Table S4 The sequence of primers for ChIP**

| primer 1 | Forward:5′-GGTCTCACTCTTTCACCCAAGCT-3′ |
| --- | --- |
|  | Reverse:5′-CGCCCCGACTTGTCTCTCTACAAA-3′ |
| primer 2 | Forward:5′-CCTGTCTCAGCCTATTGAGTAGCT-3′ |
|  | Reverse:5′-AACACTTCAGGAGGCTGAAGAGG-3′ |
| primer 3 | Forward:5′-GGCTGGAGTGCAATGGTGTGTTCT-3′ |
|  | Reverse:5′-TTCAAGACCAGCCTGGCCAACATGA-3′ |
